# The Hidden Regulators: MicroRNAs in Pediatric Heart Development and Disease

**DOI:** 10.3390/jcm14196833

**Published:** 2025-09-26

**Authors:** Adam Kozik, Michał Piotrowski, Julia Izabela Karpierz, Mariusz Kowalewski, Jakub Batko

**Affiliations:** 1 CAROL—Cardiothoracic Anatomy Research Operative Lab, Department of Cardiovascular Surgery and Transplantology, Institute of Cardiology, Jagiellonian University Medical College, 31-008 Krakow, Poland; 2 Faculty of Medical Sciences in Katowice, Medical University of Silesia, 40-055 Katowice, Poland; 3 Department of Cardiac Surgery and Transplantology, National Medical Institute of the Ministry of the Interior and Administration, 137 Wołoska Street, 02-507 Warsaw, Poland

**Keywords:** miRNA, congenital heart disease, pediatric cardiology

## Abstract

The development and function of the heart are governed by a highly coordinated network of regulatory mechanisms, among which miRNAs play a central role. These small, non-coding molecules modulate gene expression predominantly through mRNA degradation. This narrative review aims to summarize current knowledge about biogenesis, its impact on heart development and function, and its clinical implications in pediatric cardiology. We discuss how specific miRNAs contribute to shaping the normal heart and influencing the pathogenesis of congenital malformations. Furthermore, we review disease-specific miRNA signatures identified in the most common congenital heart defects and some acquired diseases, including hypoplastic left heart syndrome (HLHS), tetralogy of Fallot (TOF), bicuspid aortic valve (BAV), septation defects, cardiomyopathies, arrhythmias, and myocarditis. Many studies indicate that circulating and tissue miRNAs can become non-invasive biomarkers for early diagnosis and disease monitoring. Experimental data suggest their potential use in treatment despite many delivery and safety challenges. However, further research is necessary to fully exploit the potential of miRNAs and effectively translate these findings into clinical practice in pediatric cardiology.

## 1. Introduction

MicroRNAs (miRNA) are small, non-coding RNA molecules, typically 19–25 nucleotides in length, that play a crucial role in the post-transcriptional regulation of gene expression. By binding to complementary sequences within target messenger RNAs (mRNAs), they can promote mRNA degradation or inhibit translation, thus influencing a wide range of biological processes. Since their discovery in the early 1990s, miRNAs have emerged as essential regulators in cardiovascular development, physiology, and pathology [1,2]. In recent years, significant progress has been made in understanding their role not only in adult cardiology but also in pediatric cardiac conditions [1].

Pediatric cardiology presents unique challenges compared to adult practice. Many cardiovascular disorders in children are congenital in origin, and their pathophysiology is often linked to disturbances in embryonic cardiac development, genetic syndromes, or early-life environmental influences [1,3]. Even cardiovascular diseases seen in adults, such as myocarditis or cardiomyopathies, can differ significantly in their clinical course and molecular mechanisms from those in children [4,5]. Therefore, biomarkers and therapeutic targets need to be explicitly adapted to the pediatric population. In this context, miRNAs represent a promising tool for diagnosis, prognosis, and potentially treatment of various cardiac diseases in children.

Alterations in miRNA expression patterns have been reported in several types of CHDs, with great heterogeneity between different CHDs and even between treatment stages [6,7,8,9,10,11,12].

Beyond congenital conditions, miRNAs are also involved in pediatric acquired cardiac diseases. For example, distinct circulating miRNA signatures have been reported in children with myocarditis [13,14,15,16,17]. In some cases, these miRNA patterns correlate with disease severity, ventricular function, or treatment response, suggesting their potential utility as non-invasive biomarkers. Furthermore, miRNAs have been implicated in cardiac remodeling, arrhythmogenesis, and heart failure progression in young patients, making them relevant for long-term disease monitoring.

This study is a narrative review that synthesizes current evidence on the miRNAs’ role in diagnosis, treatment, and understanding of various pediatric cardiac conditions. The review does not adhere to a registered protocol and does not employ a systematic search strategy. Instead, articles were selected through targeted searches of major databases (MEDLINE, Embase, Scopus, Google Scholar) and manual review of reference lists, with an emphasis on clinically relevant and recent studies. Consequently, this review may not include all available publications on the subject, and its findings should be interpreted in light of these methodological limitations.

## 2. Biology of MicroRNA: Mechanisms and Functions

### 2.1. miRNA Biogenesis

Over the past three decades, since the teams of Victor Ambros and Gary Ruvkin published groundbreaking papers first describing the functions of what was later recognized as encoding miRNAs, our understanding of these small regulatory RNAs has advanced substantially [18,19,20]. Advances in research have allowed the identification of several miRNA biogenesis pathways, which can be broadly classified into canonical and non-canonical [21]. In the canonical pathway, transcription is carried out by RNA polymerase II (Pol II). The primary transcript, known as a pri-miRNA, contains a characteristic hairpin-like structure [22]. This feature is essential for recognition by the Microprocessor complex, which consists of the Drosha endonuclease with RNase III activity and its two partner proteins, DGCR8. The Microprocessor cleaves both strands of the hairpin stem, releasing a precursor molecule called pre-miRNA [23].

**Figure 1:**
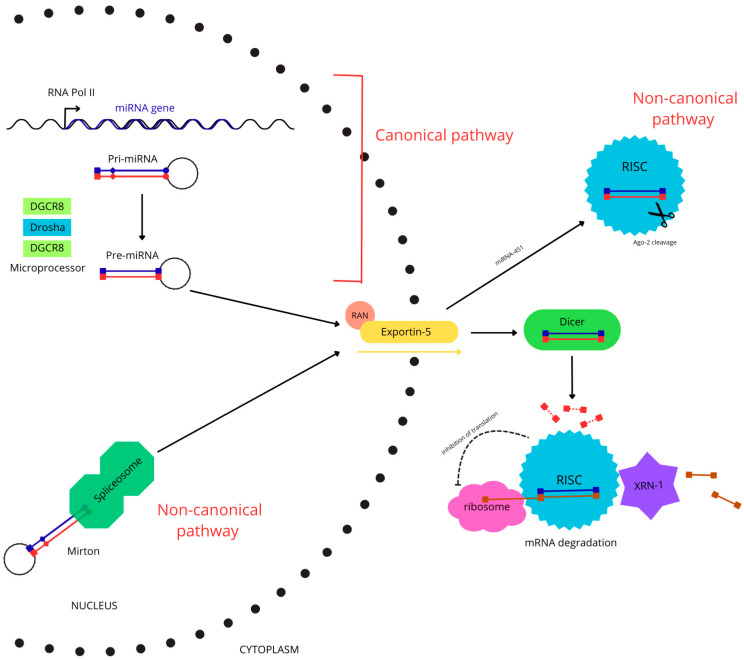
miRNA biogenesis and mechanisms of action.

This nuclear processing is followed by cytoplasmic maturation. Pre-miRNA is exported via the activity of Exportin 5 and RAN-GTP proteins [24]. Then, the transcript is processed by Dicer—a protein that, like Drosha, possesses RNase III domains, allowing for further cleavage near the loop and the formation of a duplex consisting of the miRNA strand and a complementary “passenger strand” [25]. In the next step, the duplex binds to proteins from the Argonaute (Ago) family, supported by the chaperones HSC70 and HSP90 [26]. One strand is degraded, and the other becomes a fully mature miRNA, approximately 22 nucleotides long. The pU or pA content at the 5’ region determines which of the strands is retained, due to specific interactions with the MID domain of the Ago2 protein [27]. MiRNA bound to Ago proteins forms an RNA-induced silencing complex (RISC), which can recognize and lead to the degradation of target mRNAs [28]. Most miRNAs are produced as a result of the process described above, but there are also several non-canonical synthesis pathways that bypass some of the steps or utilize unusual precursors (Figure 1).

In the canonical pathway, after transcription by Pol II, the pri-miRNA forms a hairpin-like structure, which is cleaved by the Microprocessor. The released pre-miRNA is transported from the nucleus to the cytosol by Exportin 5 and RAN-GTP. After cleavage by Dicer, a ~22 bp miRNA duplex is formed, and one of the strands is degraded. The mature miRNA, when bound to the Argonaute protein (Ago), forms a silencing complex (RISC). In the less common non-canonical pathways, the requirement for Microprocessor or Dicer cleavage can be bypassed. In the case of so-called mirtrons, excision of an intron by the spliceosome directly generates a pre-miRNA. The transcript bypasses the Microprocessor complex and is immediately exported to the cytoplasm, where it undergoes further processing by Dicer. In contrast, miRNA-451 maturation requires Drosha, but not Dicer activity. Its nuclear processing is similar to that of canonical miRNAs. However, once exported to the cytoplasm, pre-miRNA-451 binds directly to Ago, whose catalytic activity generates the mature miRNA. Upon recognition of a target mRNA, the RISC can trigger its degradation through the action of the exonuclease XRN-1. Post-transcriptional inhibition may also occur through interactions of the RISC with the ribosome.

For example, mirtrons produced by transcription of some introns are immediately exported to the cytosol, bypassing cleavage by Drosha [29]. Other miRNAs, such as miR-451, are Dicer-independent, and the final product is formed by cleavage of the 3’ arm of the hairpin by Argo2 protein [30]. Understanding the subsequent stages of transcript maturation opens new possibilities for the development of synthetic miRNAs with therapeutic potential.

### 2.2. Regulation of miRNA Expression and Decay

Most miRNAs originate from introns or non-coding transcripts, while a minor fraction overlaps with exons of protein-coding genes [21]. Their expression can be regulated at multiple levels, including transcription, maturation, or degradation of the final product. Pol II regulates the transcription process in a manner analogous to that of protein-coding genes for all canonical and most non-canonical miRNAs [31]. In addition, numerous mechanisms modulate the activity of pivotal enzymes such as Drosha or Dicer [32]. Post-transcriptional modifications of pri- or pre-miRNAs represent another essential layer of regulation. One of the best-characterized examples is Lin-28-mediated oligo(U) tailing of the let-7 family, which blocks Dicer processing [33]. Notably, even single-nucleotide changes can sometimes be crucial. For instance, 3′-end monouridylation exerts the opposite effect, enhancing maturation [34]. Likewise, adenosine deamination can trigger pri-miRNA degradation by adenosine deaminases acting on RNA (ADARs) [35]. Additionally, many RNA-binding proteins, such as hnRNP A1 or SRSF1, can regulate the activity of specific miRNAs through diverse mechanisms [36].

The biological functions of miRNAs are mainly dependent on their stability. Most have biological half-lives of several hours, but there are many exceptions [37]. For example, in retinal photoreceptor cells, the levels of many miRNAs change dynamically in response to light stimuli [38,39]. One of the key mechanisms responsible for this phenomenon is target-directed miRNA degradation (TDMD). The binding of an mRNA to sequences at the miRNA 3′ end, distinct from classical miRNA-RNA interactions that typically involve the seed region at the miRNA 5′ end, triggers the recruitment of the ZSWIM8 ubiquitin ligase. This, in turn, promotes miRNA ubiquitination and degradation via the action of specific exonucleases [40].

### 2.3. Mechanism of Action

Although Ago proteins within the RISC possess exoribonucleolytic activity and contain a PIWI domain with a catalytic center resembling RNase H, degradation of target mRNAs is rarely the result of direct cleavage [41,42,43]. Upon mRNA binding to the RISC, additional proteins such as TNRC6 are recruited. Their interaction with the poly(A)-binding protein (PABPC) leads to the activation of protein complexes with deadenylase activity, among which the CCR4-NOT complex plays a central role [44,45]. Poly(A) tail shortening weakens PABPC interactions with the translation initiation factors eIF4E and eIF4G, exposing the 5′ cap structure to the Dcp2 decapping enzyme. Removal of the cap renders the transcript susceptible to 5′→3′ degradation by the Xrn1 exonuclease [46]. While mRNA destabilization is the primary mechanism responsible for miRNA-mediated repression, interactions between RISC components and specific protein partners can also directly inhibit translation [47].

Target recognition by miRNAs most often relies on binding sites that promote TNRC6-dependent repression and span nucleotides 2–7 of the miRNA (the so-called seed region), usually extended by two additional nucleotides, and located within the 3′UTR of the mRNA [48]. In some cases, 3′-compensatory sites can also be utilized, which bind to extensive sequences at the 3′ end of the mRNA in the case of an incomplete seed region match. Other non-canonical binding sites have also been described; however, these typically involve short stretches of complementarity, and seed region pairing remains essential for efficient translational repression [21].

## 3. Role of miRNA in Cardiac Development and Physiology

MiRNA, by regulating gene expression, plays a key role in prenatal development [49]. In recent years, its role in cardiogenesis and its influence on the occurrence of heart defects have been increasingly emphasized [50]. For example, deletion of the Dicer enzyme in mice, which is essential for the maturation of canonical miRNAs, led to disruption of heart septation and prenatal death [51]. In turn, its cardiac-restricted deletion in 3-week-old mice resulted in the development of lethal dilated cardiomyopathy [52]. This suggests that miRNA is essential not only for cardiogenesis but also for the maturation and proper functioning of this organ [2]. Moreover, changes in miRNA expression in many cardiovascular diseases indicate their role in maintaining adequate homeostasis and provide hope for the development of new therapies and diagnostic methods.

To better understand the role of miRNA in cardiogenesis, it is worth emphasizing that its levels vary between different tissues. It is estimated that the majority, as much as 45%, of miRNAs in the rodent heart are miR-1 [53]. Several other miRNAs, termed MyomiRs, have also been identified in large numbers in the developing heart. These include miR-133 and miR-206, miR-143 and miR-208 [50]. The functions of some key miRNAs involved in development are summarized in Table 1.

### 3.1. miRNA as a Regulator of Cardiomyocyte Maturation

A fundamental role in cardiogenesis is played by the specification of cardiac mesoderm under the influence of growth factors such as bone morphogenetic protein (BMP), fibroblast growth factor (FGF), and wingless-type (WNT) protein family [50]. Central to this process are miR-1 and miR-133, which originate from two genomic clusters: miR-1/133a-1 on chromosome 18 and miR-1/133a-2 on chromosome 20 [54].

MiR-133 along with miR-499 influences the expression of transcription factors such as GATA4 and Nkx2.5, and cardiac proteins like connexin 43 (Cx43) and cardiac troponin T (cTnT), with both miRNAs exhibiting synergistic effects [55].

Moreover, miR-499 plays an essential role in remodeling type II (fast) muscle fibers toward type I (slow) fibers. Along with miR-208b, encoded in the intron of the myosin heavy chain gene, it is critical for modulating its expression by suppressing transcriptional repressors such as Sox6, Purβ, and Sp3 [56].

A promising potential biomarker is miR-20b-5p, acting as an inhibitor of cardiac cell differentiation [57]. Its elevated levels have been observed in patients with atrial septal defect (ASD). Its mechanism involves inhibition of transcription factors such as the GATA4 above and Nkx2.5 via repression of Tet methylcytosine dioxygenase 2 (TET2)—an enzyme catalyzing epigenetic modifications that promote transcription [57]. Alongside this, miR-128a plays a regulatory role in heart development, modulating the differentiation of cardiac precursor cell populations. It likely acts as a switch that determines the direction of differentiation of pluripotent cells—whether they become cardiomyocytes or cells of the conduction system [58].

### 3.2. miRNA as a Regulator of Proliferation and Cell Cycle

Alongside miR-1, one of the most critical proliferation inhibitors is the miR-15 family. Expression of miR-15a and miR-16-1 increases between days 1 and 9 after birth in mice, leading to the inhibition of a broad spectrum of cell cycle-related genes such as Chek1, Cdc25A, Ccnd1, and Ccne1 [59].

In contrast, the miR-302-367 cluster, highly expressed in embryonic stem cells, promotes cell division and is essential for maintaining pluripotency [60]. It acts similarly to the miR-17-92 cluster, which inhibits key genes for cell cycle progression such as Cdkn1a, Rb1, and E2F1, and whose function is essential even during postnatal development [61]. MiR-410 and miR-495 also play an important role in stimulating cardiomyocyte proliferation at the prenatal stage. Both act by targeting the transcriptional coactivator Cited2 [62]. Another factor supporting proliferation is the miR-106b~25 cluster, which is highly expressed in mice during the first two weeks after birth. It inhibits numerous cell cycle inhibitors, such as Cdkn1a and Wee1. Loss of its function manifested as mild but significant eccentric hypertrophy and reduced ejection fraction, suggesting this cluster maintains the balance between cardiomyocyte hypertrophy and hyperplasia [63]. Additionally, a high-throughput functional screening study identified miR-515-3p and miR-519e-3p as potent inducers of proliferation in cardiomyocytes derived from human induced pluripotent stem cells (hiPSC-CMs) [64].

### 3.3. miRNA as a Regulator of Angiogenesis

Adequate vascularization of the myocardium plays a critical role in supporting its regeneration after infarction, making the identification of miRNAs influencing this process crucial for developing new therapeutic strategies. One of the most important promoters of angiogenesis is miR-126, which enhances the endothelial response to VEGF by suppressing endogenous inhibitors of this pathway (SPRED1 and PIK3R2) by directly inhibiting their translation [65]. In response to hypoxia, miR-210, which plays a similar role, is activated [66]. Conversely, miR-92a acts as an inhibitor, and its suppression promotes neovascularization following myocardial ischemia [67]. This insight led to the development of a synthetic microRNA-92a inhibitor (MRG-110) aimed at promoting wound healing in both diabetic and non-diabetic wounds [68]. Notably, a phase I clinical trial evaluating the safety of MRG-110 following intradermal administration in healthy volunteers was completed in 2019 (NCT03603431). Another significant cluster is miR-143/145. It regulates the ability of VSMCs to adopt a communicative contractile phenotype by suppressing ACE expression. Their loss leads to chronic activation of the RAA system, contractile dysfunction, and the initiation of neointimal changes, making them a promising target for the treatment of vascular damage [69].

### 3.4. miRNA as a Regulator of the Cardiac Conduction System

The cardiac conduction system (CCS) is crucial for the generation and conduction of impulses that initiate myocardial contraction. Its most important components include the sinoatrial node (SAN), which serves as the primary pacemaker, the atrioventricular node, the His bundle, its branches, and a network of Purkinje fibers [70]. miRNAs play essential roles in their development. Deletion of miR-17-92 and miR-106b-25 was found to lead to increased expression of the Shox2 and Tbx3 genes, key players in SAN development. Loss of function of these clusters significantly increased the risk of developing atrial fibrillation (AF) in mice [71]. Many miRNAs also play a role in regulating the function of specific ion channels, e.g., HCN (hyperpolarization-activated cyclic nucleotide-gated), which are crucial for the pacemaker function of SAN [72]. miR-486-3p has been shown to inhibit HCN4 expression, leading to a decrease in heart rate. The same receptor is also acted on by miR-370-3p, whose silencing in heart failure can restore normal pacemaker function. [73]. Furthermore, miR-423-5p regulates HCN4 in the context of bradycardia induced by regular sports training [74]. The previously mentioned miR-1 also influences the expression of KCNJ2, KCND2, and GJA1 channels, and together with miR-133 regulates calcium cycling by modulating ryanodine receptor (RyR2) function [2,75,76]. Additionally, miR-208a is crucial for the expression of some connexins, proteins necessary for proper conductance [77].

**Table 1:** Function of selected miRNAs in cardiac development.

microRNA	Primary Function	Targeted Genes/Pathways	Effect of Dysregulation	References
miR-1	Inhibition of the cell cycle	Myocardin, WNT, FGF	Knockout: dilated cardiomyopathy due to sarcomere disruptions and postnatal lethality (murine)	[78,79]
miR-133	Anti-apoptotic protection and synergistic effects with miR-499	GATA4, Nkx2.5, Cx43, cTnT	Knockout: dilated cardiomyopathy, VSD, fibrosis (murine)	[55]
miR-499	Muscle fiber remodeling (type II→type I) and cardiomyocyte differentiation	Sox6, Purβ, Sp3, GATA4, Nkx2.5, Cx43, cTnT	Overexpression: conversion of muscle fibers to slow type (murine)	[55,56]
miR-208b	Muscle fiber remodeling and myosin heavy chain expression	Sox6, Purβ, Sp3, myosin heavy chain gene	Knockout: inhibition of the formation of slow muscle fibers (murine)	[56,80]
miR-20b-5p	Inhibition of cardiac cell differentiation	TET2, GATA4, Nkx2.5	Knockout: more cTnT-positive cells Overexpression: fewer cTnT-positive cells (hESCs-CMs)	[57]
miR-128a	Regulation of differentiation, guidance toward a cardiomyocyte or conduction system phenotype	Cardiac precursor cell differentiation pathways	Knockout: slower heart rate, diminished ventricular size (zebrafish)	[58]
miR-15a	Inhibition of proliferation	Chek1, Cdc25A, Ccnd1, Ccne1	Knockout: increased number of cells entering mitosis, with cell cycle arrest (murine)	[59]
miR-16-1				
miR-17-92	Progression of the cell cycle	Cdkn1a, Rb1, E2F1		[61]
miR-106b~25	Regulation of the hyperplasia–hypertrophy balance	E2f5, Cdkn1c, Ccne1, Wee1, Hand2, Mef2d	Knockout: mild eccentric hypertrophy and reduced ejection fraction (murine) Overexpression: hyperplasia, increased proliferation (murine)	[63]

## 4. Clinical Applications of MicroRNA in Pediatric Cardiology

### 4.1. miRNA as Potential Biomarkers for Congenital Heart Disease

Multiple studies aiming to identify potential prenatal biomarkers of congenital heart diseases (CHDs) were conducted in recent years as a possible solution for the limitations of prenatal ultrasound [17,81,82,83,84]. As stated before, miRNAs play a crucial role in the development of the cardiovascular system, and alterations in their expression patterns have been implicated in the pathogenesis of congenital heart diseases. Several studies have explored whether specific miRNA profiles in maternal or fetal blood could serve as non-invasive biomarkers for prenatal CHD detection. Zhu et al. reported that circulating miR-19b, miR-22, miR-29c, and miR-375 were significantly elevated in pregnant women carrying fetuses with CHD, achieving good diagnostic accuracy [85]. Similarly, Gu et al. identified a panel including miR-142-5p, miR-1275, miR-4666a-3p, and miR-3664-3p that effectively distinguished CHD cases from healthy controls [86]. Beyond diagnostic associations, mechanistic insights were provided by studies on miR-29b-3p, which was found to be increased in the right ventricular outflow tract of CHD patients [87]. Experimental overexpression of this miRNA in zebrafish embryos caused developmental delay, cardiac malformations, and impaired function, while its inhibition promoted cardiomyocyte proliferation. This effect was shown to involve direct targeting of NOTCH2, a pathway relevant to cardiac development. Kong et al. demonstrated that miR-1, miR-208, and miR-499 were reduced in umbilical cord blood from CHD fetuses, with distinct expression trends depending on the CHD subtype. These miRNAs showed good discriminatory potential for differentiating affected from healthy fetuses [88]. Other studies primarily focused on differences in miRNA expression patterns without directly assessing their diagnostic performance. For example, Yang et al. reported upregulation of miR-1-3p, miR-184, and miR-206, while Kehler et al. described increased expression of miR-99a [81,89]. Similarly, Jin et al. and others identified additional miRNAs associated with CHD [84]. Collectively, these findings further support the concept that circulating miRNAs may serve as clinically relevant biomarkers, even though their diagnostic potential remains to be fully explored. The mentioned studies suggest that specific miRNAs—such as miR-29b-3p, miR-1, miR-208, and miR-499—may serve as promising non-invasive biomarkers for the early detection of CHD, with potential applications in prenatal screening programs. Its clinical significance and possibility to introduce it into everyday practice are still to be investigated in the future before the development of specific test panels. Current literature data indicate that miRNAs are promising biomarkers for congenital heart diseases. However, significant heterogeneity across published studies must be taken into account. Differences in sample sizes, patient populations, and analytical methods hinder their clinical applicability [90]. It is worth noting that the studies cited above examined various types of materials, such as maternal or umbilical blood or heart tissue samples [85,86,87,88]. These limitations highlight the need for larger, standardized, multicenter investigations before clinical implementation can be considered.

### 4.2. miRNAs in Congenital Heart Diseases

CHDs were traditionally classified into cyanotic and non-cyanotic forms, depending on whether the defect leads to mixing of oxygenated and deoxygenated blood with resultant systemic desaturation [91,92]. Common cyanotic lesions include TOF, transposition of the great arteries (TGA), truncus arteriosus (TA), and total anomalous pulmonary venous return. In contrast, typical non-cyanotic CHDs include ventricular septal defect (VSD), atrial septal defect (ASD), patent ductus arteriosus (PDA), and coarctation of the aorta (CoA). Beyond differences in clinical presentation, prognosis, and therapeutic strategies, emerging evidence suggests that cyanotic and non-cyanotic CHDs also exhibit distinct molecular signatures, including differential miRNA expression.

In one study, the expression levels of miR-21-5p, miR-15-5p, miR-221-3p, and miR-26-5p were found to differ between children with CHD and healthy controls significantly. Notably, miR-21-5p levels were particularly elevated in patients with cyanotic CHD compared to those with non-cyanotic defects. These findings suggest that specific miRNA dysregulation may contribute not only to the pathogenesis of cardiac malformations but also to the variable severity observed across CHD subtypes. The pronounced upregulation of miR-21-5p in cyanotic CHD, for instance, may reflect its association with greater disease burden and adverse clinical course [93].

More recent classification systems take into account hemodynamic and anatomical complexities, providing a framework that is more clinically relevant. To date, no studies have examined miRNA profiles in the context of these modern classification systems.

Further studies concerning those differences, especially during prenatal time, might be beneficial for early detection of those conditions and prediction of their severity. Below, we discussed current knowledge about the molecular profiles of certain CHDs [93].

#### 4.2.1. Hypoplastic Left Heart Syndrome

Univentricular heart is one of the most severe forms of congenital heart disease, characterized by underdevelopment of the cardiac structures and functional dependence on one ventricle [94]. HLHS is considered one of the deadliest of those conditions [95].

Several studies have explored circulating miRNAs as potential biomarkers of heart failure in patients with univentricular hearts. Abu-Halima and colleagues analyzed blood samples from 48 patients and 32 controls using a large panel of miRNA arrays, identifying 50 significantly dysregulated miRNAs [7]. Among these, miR-150-5p demonstrated the strongest association with heart failure severity, emerging as an independent predictor of overt heart failure. Importantly, its diagnostic performance was comparable to established clinical parameters such as NT-proBNP levels and NYHA functional class, and when combined with NT-proBNP, predictive accuracy was further enhanced [7]. In a complementary line of investigation, Ramachandran and colleagues focused on children with univentricular hearts, assessing whether circulating microvesicle-derived miRNAs could provide insights into heart failure progression [8]. Their findings pointed to miR-129-5p, which was inversely correlated with heart failure severity as measured by the Ross score. Experimental validation in cardiomyocyte models further suggested a mechanistic link between oxidative stress, miR-129-5p downregulation, and altered expression of bone morphogenetic protein receptor 2, a pathway implicated in pulmonary vascular disease [8]. Together, these studies highlight that distinct miRNAs—miR-150-5p and miR-129-5p—hold promise as non-invasive biomarkers in univentricular hearts, potentially offering additive value to traditional measures for risk stratification and disease monitoring. While Abu-Halima emphasized their prognostic utility in predicting overt heart failure, Ramachandran provided mechanistic insights linking miRNA regulation to stress responses in pediatric cardiomyocytes. These complementary perspectives underscore the potential of miRNA profiling to refine biomarker strategies in HLHS.

Beyond circulating biomarkers, studies of myocardial tissue have provided complementary insights into the role of miRNAs in univentricular physiology. Suharov and colleagues examined miRNA expression directly in right ventricle (RV) tissue from children with HLHS and demonstrated that the miRNA profile in this setting was essentially distinct from that observed in adult idiopathic dilated cardiomyopathy, underscoring the uniqueness of developmental mechanisms in congenital heart disease [9]. Dysregulated miRNAs were found to target several transcription factors and signaling pathways fundamental for cardiac morphogenesis, with inverse patterns between miRNA levels and the expression of their predicted targets [9]. Notably, the myocardial miRNA signature was not static but evolved across the stages of surgical palliation. Analysis of the miRNA expression in patients with HLHS revealed significant differences related to right ventricular volume overload. The most noteworthy finding was a normalization of miR-100, miR-99a, and miR-145 levels in patients after stage 3 of surgical palliation (following the Fontan procedure) compared to those without surgery or after stage 1 (the Norwood procedure), when RV volume overload is significant. These dynamic changes suggest that the reduction in RV volume load achieved later in palliation can partially reverse miRNA dysregulation. In contrast, the expression of miR-204 and miR-137-3p remained altered regardless of the stage of surgical treatment. Changes in miR-204 expression were also observed in other patients with idiopathic dilated cardiomyopathy, which may indicate its potential use in pediatric heart failure regardless of etiology [9]. Predicted targets included key transcriptional regulators such as QKI, CDK6, SOX11, FOG2, GATA6, GATA4, and dHAND, several of which showed stage-dependent expression shifts. Together, these findings indicate that some miRNA alterations reflect adaptive mechanisms in response to hemodynamic stress, while others may be linked to intrinsic defects in cardiac development [9].

As HLHS is one of the most fatal and complex CHDs, a better understanding of its pathophysiology is needed to possibly identify prenatal biomarkers and investigate whether specific miRNA profiles are related to hemodynamic and clinical outcomes of surgical treatment of the disease.

#### 4.2.2. Tetralogy of Fallot

Among cyanotic congenital heart diseases, TOF occupies a central role, representing the most frequent lesion within this group. Epidemiological studies indicate that TOF constitutes roughly 5–7% of all congenital cardiac malformations. The condition arises from an abnormal conotruncal development of the heart and is classically described by four interrelated anatomic abnormalities: an anteriorly malaligned ventricular septal defect, overriding of the aortic root, obstruction of the right ventricular outflow tract (subpulmonary and/or valvular), and secondary hypertrophy of the right ventricle. The clinical spectrum is heterogeneous. In the neonatal period, the extent of right ventricular outflow tract obstruction largely determines symptomatology, with severe forms manifesting as profound cyanosis shortly after birth, while milder cases may present later in infancy. Importantly, the hypertrophic response of the right ventricle is not a primary malformation but a physiological consequence of chronic pressure overload [96].

Modern diagnostic pathways have shifted substantially with the introduction of fetal echocardiography, which allows TOF to be recognized before birth and facilitates parental counseling as well as delivery planning in tertiary care centers. In postnatal life, prompt recognition of critical right ventricular outflow tract obstruction and stabilization of affected neonates remain essential to avert hypoxemic crises and cardiovascular decompensation [97]. Current perspectives on management emphasize not only the technical aspects of surgical and catheter-based interventions but also the role of coordinated, multidisciplinary care teams. Optimal outcomes are increasingly linked to timely diagnosis, individualized treatment planning, and effective interprofessional collaboration. Because of the relatively high incidence of TOF compared to other cyanotic CHDs, the miRNA profiles in that condition were thoroughly investigated. For example, Zhu and colleagues found that, in particular maternal circulating miR-22 was upregulated in the case of TOF [85].

One of the earliest comprehensive efforts to profile non-coding RNA signatures in TOF myocardium was carried out by O’Brien and colleagues, who examined RV tissue from infants with nonsyndromic TOF compared with both fetal and typically developing hearts [98]. Their analysis revealed marked changes in both miRNAs and snoRNAs, with expression patterns in TOF myocardium resembling those of fetal samples, suggesting a persistence of developmental transcriptional programs. A subset of 44 genes essential to cardiac development showed altered expression, correlating inversely with 33 deregulated miRNAs. These findings not only highlight potential regulatory networks disrupted in TOF but also point toward aberrant RNA processing, as evidenced by the association between snoRNA dysregulation, spliceosomal variants, and heart development. Expanding on this line, Bittel and colleagues placed particular focus on miR-421, which was significantly upregulated in TOF myocardium and negatively correlated with SOX4, a Notch pathway regulator [99]. Functional experiments confirmed its capacity to influence cardiomyocyte proliferation, positioning miR-421 as a candidate mediator of RV remodeling in TOF. Independent work by Zhang and colleagues identified 18 miRNAs with significantly altered expression in RVOT tissue, among them miR-424 and miR-222, both of which were experimentally shown to modulate cardiomyocyte proliferation and differentiation [100]. Similarly, Liang and colleagues identified miR-940 as one of the most profoundly downregulated miRNAs in TOF myocardium, particularly enriched in the normal RVOT. Functional assays demonstrated that miR-940 loss enhanced progenitor proliferation while impairing migration via direct regulation of JARID2, thus linking altered secondary heart field progenitor dynamics with TOF development [101]. Other groups have focused on well-characterized cardiac miRNAs [102,103]. Wu reported that miR-1 and miR-206 were downregulated in TOF myocardium, resulting in increased expression of connexin-43, a protein crucial for myocardial conduction [102]. Grunert further integrated genome-wide miRNA and mRNA datasets in TOF myocardium, identifying key regulatory axes including miR-1/miR-133 pairs acting on structural and electrophysiological genes such as KCNJ2 and TNNI1 [103]. Collectively, these studies suggest that while many miRNAs in TOF reflect fetal-like transcriptional states, others directly perturb pathways critical to RV proliferation, conduction, and morphogenesis.

The contribution of miRNAs is not restricted to myocardial tissue. Circulating miRNA profiles in patients late after surgical repair TOF repair were systematically explored by Abu-Halima, who reported broad dysregulation across dozens of miRNAs, with several (miR-181d-5p, miR-206, miR-625-5p) showing excellent discriminatory value between TOF patients and controls [6]. Interestingly, miR-421 and miR-625-5p were reduced explicitly in individuals who developed symptomatic right heart failure, underscoring the potential prognostic role of circulating miRNAs in disease progression [6].

Complementary to this, Gomez and colleagues identified increased expression of miR-221-5p, miR-21-5p, and miR-155-5p in children with TOF [104]. Target predictions implicated these miRNAs in hypoxia-sensitive signaling pathways, including AKT, SMAD, and TNF-α, supporting the concept that circulating miRNAs reflect both developmental abnormalities and secondary remodeling processes. More recently, Yang and colleagues analyzed exosomal miRNAs in amniotic fluid from fetuses diagnosed with TOF [105]. Over 250 miRNAs were differentially expressed, with enrichment in Notch and Wnt signaling pathways. Functional experiments highlighted miR-10a-5p as a suppressor of TBX5, a master transcription factor in cardiogenesis, thus providing a possible mechanistic link between prenatal exosomal signaling and impaired cardiomyocyte differentiation. Beyond tissue and circulating profiles, genomic approaches have expanded the picture of miRNA involvement in TOF. Bassett and colleagues analyzed the role of copy number variants (CNVs) overlapping miRNA loci, demonstrating an enrichment of rare CNVs in TOF patients independent of the classic 22q11.2 deletion [106]. This supports a multi-hit model in which structural variation affecting miRNA dosage may contribute to susceptibility. At the systems level, integrative bioinformatics analyses by You and colleagues constructed miRNA–mRNA–TF co-regulatory networks, identifying hub genes and pathways spanning ubiquitin-mediated proteolysis, ribosomal function, and energy metabolism [107]. Such work provides a higher-order view of how diverse miRNA alterations converge on essential developmental and metabolic processes in TOF. Adding another layer, Wang and colleagues applied high-throughput sequencing and uncovered sex-specific differences in small RNA expression, with miR-1/133 accounting for the most significant variance between male and female TOF hearts [108]. This observation raises the possibility that miRNA regulation may partly explain reported sex differences in CHD prevalence and outcomes.

#### 4.2.3. Septation Defects

VSD and ASD are the two most common CHDs, with incidences of 0.03% of newborns in the case of VSD and 0.016% in the case of ASD [109,110]. Prenatal diagnosis of ASD and VSD was investigated by Zhu and colleagues and discussed beforehand.

##### Ventricular Septal Defect

VSD accounts for nearly 40% of all congenital cardiac malformations and frequently requires surgical intervention [110]. While their etiology is multifactorial, encompassing both genetic and environmental influences, accumulating evidence points to an essential role of miRNAs in their pathogenesis. In an early investigation, Li and colleagues analyzed 25 candidate miRNAs in cardiac tissue from patients with congenital heart disease and healthy controls. They reported that miR-1-1 and miR-181c exhibited disease-specific alterations in individuals with VSD. Reduced miR-1-1 levels were associated with increased expression of GJA1 and SOX9, while elevated miR-181c correlated with downregulation of BMPR2. Functional validation confirmed direct interactions between these miRNAs and their predicted targets, implicating them in septal morphogenesis and suggesting a mechanistic contribution to VSD development [10]. The same group later extended their work to circulating miRNAs. In plasma samples from children with VSD, they identified 36 dysregulated miRNAs, of which RT-qPCR validated eight. Bioinformatic analysis pointed to regulatory networks involving key developmental genes such as NOTCH1, HAND1, ZFPM2, and GATA3. This study provided the first evidence that peripheral miRNA profiles reflect underlying cardiac developmental disturbances, and highlighted their potential as non-invasive biomarkers for VSD [111]. The clinical translation of these findings was further advanced by Gu et al., who investigated maternal serum from pregnancies affected by congenital heart disease. They identified 38 dysregulated circulating miRNAs, with 12 validated by real-time PCR. A biomarker panel consisting of four miRNAs—miR-142-5p, miR-1275, miR-4666a-3p, and miR-3664-3p—demonstrated excellent diagnostic accuracy, achieving an AUC of 0.92 for distinguishing affected pregnancies from controls. Significantly, several miRNAs declined rapidly after delivery, confirming their fetal origin and supporting their utility as non-invasive prenatal diagnostic tools [86]. More recently, attention has shifted to exosome-derived miRNAs, which may provide more stable and specific biomarkers. Jin et al. profiled maternal circulating exosomal miRNAs in pregnancies with fetal VSD and reported 77 differentially expressed candidates. Five were validated, among which hsa-miR-146a-5p emerged as an exceptionally robust biomarker, achieving near-perfect diagnostic performance with an AUC of 0.997 [84].

##### Atrial Septal Defect

Research into the role of miRNAs in ASD has gradually evolved from genetic association studies to functional and mechanistic insights. The first evidence came from genetic analyses. Yu and colleagues identified a single-nucleotide polymorphism in miR-196a2 (rs11614913 T>C) that was associated with ASD susceptibility [112]. Carriers of the C allele demonstrated a reduced risk of developing ASD, and functional assays suggested that the variant modulated miRNA expression and its influence on cell differentiation and morphogenesis. In the same year, Wang and colleagues described a familial form of ostium secundum ASD II linked to a mutation in the 3′UTR of the ACTC1 gene (c.*1784T>C) [113]. This variant created a de novo binding site for miR-139-5p, leading to pathological repression of ACTC1. Significantly, the effect could be intensified by miR-139-5p mimics and alleviated with inhibitors, demonstrating a direct gain-of-function mechanism where altered miRNA–mRNA interaction contributes to septal malformation.

A shift towards circulating biomarkers followed. Song and colleagues reported that children with ASD had elevated levels of hsa-let-7a, hsa-let-7b, and hsa-miR-486 [11]. Among these, let-7a and let-7b were specific to ASD compared to other septal defects. Remarkably, the same miRNA changes were detectable in the mothers of affected children, suggesting potential use in prenatal screening. Receiver-operating characteristic analyses confirmed their diagnostic accuracy, highlighting circulating and maternal miRNAs as promising non-invasive biomarkers for ASD. At the tissue level, Han and colleagues performed a systematic profiling of atrial septa from ASD fetuses [114]. They identified 70 dysregulated miRNAs, with consistent upregulation of the miR-29 and miR-143/145 clusters, and downregulation of the miR-17-92, miR-106b-25, and miR-503/424 clusters. Mouse model validation showed that all except miR-143/145 were functionally associated with septal defects, pointing to cluster-level dysregulation as a driver of abnormal atrial septation.

Most recently, mechanistic insights into transcriptional regulation have emerged. Li et al. studied a family with a novel splicing mutation in NKX2-5 (c.335-1G>A) [115]. Patient-derived hiPSC cardiomyocytes showed that NKX2-5 haploinsufficiency disrupted apoptosis and proliferation during cardiomyocyte development. Crucially, these effects were mediated through the regulation of miR-19a/b, positioning miRNAs as central downstream effectors of transcription factor dysfunction in ASD [12].

##### Atrioventricular Defects

On the other hand, atrioventricular defects are relatively less common, constituting from 3 to 7 percent of CHDs. Those defects are often seen in patients with Down Syndrome—40 to 50% of them have this type of septation defect. Therefore, it was proven that miRNAs strictly linked to Down syndrome (found on chromosome 21) are expressed in cardiac tissue of patients with atrioventricular defect [116]. Among them, the miR-99a/let-7c cluster appears to play a key role. MiRNAs within this cluster influence cell differentiation in the early stages of cardiogenesis. Increased levels of miR-99a/let-7c and decreased expression of its targets, such as Ezh2 and Smarca5, have been observed in the hearts of human fetuses with trisomy 21 [117]. Moreover, in a study aimed at identifying genes and miRNAs associated with AVSD in patients with Down syndrome, Wang L et al. identified several miRNAs (miR-518a, miR-518e, miR-518f, miR-528a, and miR-96) that may contribute to the development of AVSD by regulating the expression of genes important for cardiomyocyte differentiation and adhesion (AUTS2 and KIAA2022) [118].

#### 4.2.4. Pulmonary Hypertension Secondary to Congenital Heart Disease

Pulmonary hypertension (PH) is related to a significant medical burden; it is relatively common in cases of some CHDs. Multiple studies investigating the miRNA role in secondary PH were conducted in recent years. Recent studies have highlighted the role of miRNAs as regulators, biomarkers, and potential therapeutic targets. High-throughput sequencing and bioinformatic analyses have revealed disease-specific miRNA signatures in pediatric PH, which not only correlate with invasive hemodynamic indices but also change dynamically during treatment. Circulating molecules such as miR-122-5p, miR-124-3p, miR-204-5p, and miR-9-5p have been shown to decrease with therapy, linking them to key pathways including TGF-β, VEGF, PI3K/Akt, cGMP-PKG, and HIF-1 signaling [119].

Regional profiling of miRNA gradients across the right ventricle and pulmonary circulation provided additional insight into their spatial regulation. In children with pulmonary arterial hypertension (PAH), distinct step-up and step-down patterns of miRNAs such as miR-193a-5p and miR-423-5p were observed, while others, including miR-29a-3p, miR-26a-5p, and miR-200c-3p, were upregulated compared with controls. These expression changes correlated with prognostic parameters such as pulmonary vascular resistance and RV function, suggesting that miRNA gradients may represent a novel layer of epigenetic regulation and biomarker development [120]. Several candidate miRNAs have been identified in CHD-associated PAH specifically. Elevated serum miR-27b and reduced miR-451 were independently associated with increased pulmonary pressures and biochemical markers such as BNP and ADMA, highlighting their potential combined diagnostic utility [121]. Similarly, increased circulating miR-19a distinguished CHD patients with PAH from those without, with good diagnostic accuracy, supporting its role as a clinically useful biomarker [83].

Mechanistic studies further underscore the importance of specific clusters. The miR-143/145 axis has been shown to regulate pulmonary artery smooth muscle cell migration and apoptosis, with miR-143-3p enriched in exosomes and exerting paracrine effects on endothelial cells. Inhibition of miR-143-3p ameliorated experimental PH, confirming its pathogenic role [122]. Other work demonstrated that downregulation of miR-98 in CHD-PAH correlates with pulmonary artery pressures and disease severity. As miR-98 exerts anti-hypertrophic effects in cardiomyocytes, its reduced expression may contribute to vascular remodeling, and ROC analysis indicated strong diagnostic potential [123].

Beyond individual miRNAs, circRNA-miRNA-mRNA networks are beginning to be explored. In pediatric CHD-PAH, specific circRNAs were found to be dysregulated, predicted to target miRNAs implicated in oxidative phosphorylation and endothelial junction biology, suggesting a complex regulatory axis in pulmonary vascular remodeling [124]. Lung tissue analyses confirmed broad alterations in miRNA expression, with miR-27b notably upregulated and linked to NOTCH1 regulation, further connecting it with the pathogenesis of CHD-associated PAH [125].

Recent investigations have highlighted the presence of circulating miRNA gradients across the pulmonary and cardiac vasculature in PAH. In a pediatric cohort, plasma samples were prospectively collected during cardiac catheterization from the superior vena cava, pulmonary artery, and ascending aorta in 12 children with PAH and nine matched controls. Unbiased profiling of 754 miRNAs revealed distinct trans-right-ventricle and transpulmonary gradients: miR-193a-5p and miR-423-5p exhibited opposing patterns between PAH patients and controls, while miR-26b-5p and miR-331-3p demonstrated transpulmonary differences. Additional miRNAs, including miR-29a-3p, miR-26a-5p, miR-590-5p, and miR-200c-3p, were upregulated in PAH superior vena cava samples, correlating with prognostic hemodynamic parameters [122].

In an earlier study conducted on adult patients, circulating levels of muscle-specific miR-204 were evaluated at multiple sites along the pulmonary vasculature [126]. In WHO Group I PAH, miR-204 increased sequentially across the pulmonary circulation, while intracellular miR-204 in pulmonary artery smooth muscle cells was reduced, suggesting selective excretion by diseased cells. Peripheral blood mononuclear cells showed no significant changes, highlighting the tissue-specific nature of these alterations. These findings reinforce the concept that circulating miRNA gradients reflect underlying cellular pathobiology and differ between PH subtypes, offering potential as biomarkers and therapeutic targets.

#### 4.2.5. Bicuspid Aortic Valve

BAV is the most common congenital heart defect found in adults. In some cases, it is concomitant to other CHDs, especially aortic ones [127,128]. Other hemodynamic properties characterize a standard aortic valve with three leaflets; therefore, BAV might lead to aortic root dilatation and increased shear stress on the aorta, leading to various complications [128].

There is still a limited amount of data concerning pediatric cohorts in the case of the role of miRNA in BAV. In one study, circulating miRNAs were proposed as early biomarkers of aortic remodeling. In children with BAV, serum miR-130a was significantly reduced in patients with aortic dilatation, inversely correlating with aortic diameter and implicating TGF-β signaling in disease progression. These data indicate that miR-130a can be used as a potential marker to differentiate patients into high and low risk [129].

More studies were performed in adult settings. Several circulating miRNAs have been linked to BAV-associated aortopathy. Aortic tissue profiling revealed upregulation of let-7e-5p and miR-196a-5p and downregulation of miR-17a-5p in BAV with dilatation, with correlations to HDL-C and valvular function, highlighting their biomarker potential [130]. Another study showed that miR-21, miR-133a, miR-143, and miR-145 are consistently altered in plasma and tissue, supporting their role as systemic indicators of proximal aortic wall remodeling [131]. Tissue-level investigations expanded on these findings. Whole-miRNome sequencing identified 12 dysregulated miRNAs in BAV-related thoracic aortic aneurysm, with downregulation of miR-424-3p and miR-3688-3p associated with Hippo, ErbB, TGF-β, and focal adhesion signaling [132]. Regional profiling of the ascending aorta showed differential expression of miR-128-3p, miR-150-5p, and miR-199b-5p between dilated and nondilated segments, linking miRNA regulation to wall shear stress and extracellular matrix remodeling [133]. Earlier array studies on stenotic vs. regurgitant aortic valves found reduced expression of miR-26a, miR-30b, and miR-195 in stenotic valves, with functional assays confirming roles in calcification pathways, suggesting a contribution to valvular mineralization [134]. Clinical significance, functions and changes of expression pattern in various cardiac diseases of some key miRNAs are summarized in Table 2.

### 4.3. Heart Failure

In pediatric heart failure, circulating and myocardial miRNA signatures display distinct profiles from adult heart failure. Stauffer et al. demonstrated that the miRNA profile in the hearts of children with dilated cardiomyopathy differs significantly from that of adult HF patients. They identified 17 unique miRNAs that exhibit altered or even opposite regulation in children compared to the adult population. Notably, certain miRNAs correlating with clinical responses to phosphodiesterase inhibition and Smad4 normalization, highlighting the necessity for age-specific molecular stratification. Oxidative stress–responsive miRNAs orchestrate mitochondrial integrity, antioxidant defenses, iron metabolism, ferroptosis, and survival signaling, functioning as both biomarkers and active modulators of heart failure progression. In children with advanced heart failure undergoing ventricular assist device implantation, longitudinal analyses revealed dynamic regulation of six circulating miRNAs linked to hemostatic pathways, including the downregulation of miR-409-3p, a negative regulator of coagulation factors 7 and 2, potentially reflecting a universal pro-thrombotic phenotype post-implantation [135,136,137,138].

### 4.4. Arrythmias in Children

#### 4.4.1. Supraventricular and Ventricular Arrhythmias

miR-1 and the miR-133 family stand out as regulators of cardiac excitability and conduction [139,140]. Two recent studies explored their diagnostic potential in pediatric populations presenting with either ventricular or supraventricular arrhythmias. In both studies, molecular analysis was carried out on serum samples obtained from children and adolescents diagnosed with arrhythmias, compared against healthy controls. Expression profiling focused on miR-1, miR-133a, and miR-133b [141,142]. The findings were consistent across both investigations.

Elevated levels of miR-1 and miR-133a were observed in the SVA subgroup compared to controls, highlighting a potential link between these miRNAs and supraventricular rhythm disturbances. Notably, miR-133a expression was also significantly higher in supraventricular compared with ventricular arrhythmias, suggesting subtype-specific regulation. In contrast, miR-133b was uniformly downregulated in both arrhythmic groups relative to healthy individuals, pointing to a broader role in arrhythmogenesis regardless of arrhythmia type [141,142]. miR-1 and miR-133a demonstrated moderate to strong discriminative ability in distinguishing supraventricular arrhythmia patients from arrhythmia-free controls. At the same time, miR-133b showed utility in differentiating both supraventricular and ventricular arrhythmia patients from healthy cohorts. These observations support the notion that distinct miRNA signatures may underlie specific arrhythmic mechanisms in pediatric populations [141].

Those results indicate that serum miR-1, miR-133a, and miR-133b could emerge as non-invasive biomarkers for pediatric arrhythmias. While miR-1 and miR-133a appear more closely associated with supraventricular arrhythmias, the consistent reduction in miR-133b across both arrhythmia types suggests its potential as a general marker of arrhythmic pathology. As both mentioned studies had limited sample sizes, further large-scale, multi-center studies are needed to validate these findings and to assess whether combining these miRNAs into a diagnostic panel could improve clinical accuracy.

#### 4.4.2. Long QT Syndrome

Long QT syndrome is a group of genetic disorders caused by mutations in ion channels (sodium, potassium, calcium), which could lead to lethal arrhythmias [143]. The key to its treatment was early diagnosis and prevention of those fatal rhythm disturbances either by pharmacotherapy or implantation of implantable cardioverter defibrillators in patients who were resuscitated before [143]. Studies conducted on animal models suggested the role of miR-1 and miR-133 in the development of QT-prolongation [144,145]. These miRNAs are involved in the regulation of ion channels crucial for ventricular repolarization. MiRNA-1 inhibits the expression of the GJA1 and KCNJ2 genes encoding Cx43, responsible for intracellular conductance in ventricles, and the K+ channel subunit Kir2.1, which maintains the resting membrane potential [2]. In turn, in miR-1-2 knockout mice, a decrease in KCND2 expression was observed due to the lack of negative regulation of its repressor protein Irx5. The authors concluded that loss of Irx5 may lead to disrupted ventricular repolarization, given that the KCND2 product, K+ channel subunit (Kv4.2), is responsible for the transient outward K+ current [2]. Rau et al. proved that disturbances in the biogenesis of miR-1 lead to increased expression of CACNA1C. In the case of miR-133a, it was shown to inhibit KCNQ1, a miRNA that encodes Kv7.1 [146]. It is responsible for the repolarization phase, and loss of its function leads to the development of LQTS [147]. Both miR-1 and miR-133 are responsible for increased phosphorylation of the ryanodine receptor, and their dysregulation leads to arrhythmogenesis [77]. Hendley and colleagues decided to investigate the impact of specific sequence variants of those miRNAs (miR-1 and miR-133 families) on mature miRNA products and their relation to long QT syndrome development [148]. None of the found variants affected the mature miRNA products in the investigated cohort of patients’ samples; therefore, the authors suggested another mechanism of QT prolongation in this cohort.

#### 4.4.3. miRNA as a Prognostic Tool of Arrhythmia Recurrence After Ablation

Multiple studies were conducted on the topic of miRNA changes after ablation of atrial fibrillation [149]. The ablation seems to restore certain miRNA levels to its base values; therefore, it was investigated whether they could serve as potential prognostic biomarkers of AF recurrence [149,150]. This topic was not investigated in case of pediatric cohorts nor any of the arrhythmias mentioned in this paragraph, but based on alterations of miRNA profiles in patients suffering from arrhythmias, some changes might be present post-ablation, and this topic and its clinical significance should be investigated in future studies.

### 4.5. Cardiomyopathies

miRNAs have emerged as critical post-transcriptional regulators in pediatric dilated cardiomyopathy, with roles spanning disease pathogenesis, biomarker discovery, and therapeutic innovation. Transcriptomic profiling of myocardial tissue from affected children revealed extensive age-dependent dysregulation of miRNAs, with over 390 species altered relative to non-failing controls, influencing pathways in stem cell differentiation, sarcomeric function, and intracellular signaling. Comparative analyses of paired myocardial and serum samples demonstrated that a subset of dysregulated miRNAs is consistently altered in both compartments, implicating circulating miRNAs as potential surrogates for myocardial expression changes and highlighting their involvement in processes such as inflammation, mitochondrial function, and metabolism. Prognostically, distinct circulating miRNA signatures, including upregulation of hsa-miR-155 and hsa-miR-636 and downregulation of hsa-miR-646 and hsa-miR-639, discriminate between children requiring transplantation or experiencing mortality and those with functional recovery, achieving high predictive accuracy. Therapeutically, cardiosphere-derived cells and their exosomes have shown promise in preclinical cardiomyopathy models, where exosomes enriched in cardioprotective miRNAs, notably miR-146a-5p, reduced myocardial fibrosis and improved ventricular function, findings preliminarily supported in early-phase pediatric trials. Independent profiling of plasma miRNAs has further identified distinct dysregulation patterns—including increased miR-518f and miR-454 and reduced miR-618, miR-875-3p, miR-205, miR-194, miR-302a, miR-147, and miR-544—which may represent additional diagnostic candidates for pediatric cardiomyopathy. Finally, circulating miRNA panels such as miR-142-5p, miR-143-3p, miR-27b-3p, and miR-126-3p have demonstrated diagnostic potential for childhood cardiomyopathy, with miR-126-3p and let-7g levels correlating inversely with left ventricular ejection fraction and distinguishing heart failure from non-heart failure phenotypes. Collectively, these data position miRNAs at the intersection of mechanistic insight, diagnostic precision, and emerging biologic therapy in pediatric cardiomyopathy [82,151,152,153,154].

### 4.6. Carditis

Carditis, spanning viral and rheumatic etiologies, reflects a complex convergence of myocardial injury and inflammation, with miRNAs emerging as pivotal regulatory nodes. In pediatric viral myocarditis, circulating miR-133b is markedly downregulated and inversely correlates with myocardial damage, mitigating Coxsackievirus B3–induced cardiomyocyte injury by suppressing proliferation and restraining TNF-α and IL-6 secretion via direct targeting of Rab27B. Additional miRNAs, including miR-381, miR-217, and miR-543, govern cardiomyocyte apoptosis and inflammatory signaling through modulation of COX-2 and SIRT1, whereas cardiac-associated miR-208a and miR-21 acutely mirror active injury, and subacute miR-208b predicts subsequent left ventricular functional recovery. In pediatric rheumatic carditis, miR-16-5p, miR-223-3p, and miR-92a-3p are significantly downregulated, highlighting their potential as disease-specific biomarkers. Together, these data position miRNAs as central orchestrators of inflammatory and injury pathways in carditis, offering novel opportunities for precision diagnostics and targeted therapeutics [13,14,15,16,17].

## 5. Challenges and Future Perspectives

### 5.1. Challenges in Therapeutic Applications

One of the fundamental challenges in utilizing miRNAs therapeutically is their multi-targeting nature. A single gene can be regulated by multiple miRNAs, either directly through repression of its mRNA or through the repression of relevant transcription factors. Simultaneously, a single miRNA often influences multiple genes [155]. This complexity significantly complicates the prediction of therapeutic outcomes. An example is the attempt to use miR-34a in the treatment of certain cancers. The use of a miR-34a analog resulted in serious adverse effects related to the immune response, likely resulting from the activation of two immune-related pathways [156].

Another major obstacle is the delivery of miRNAs. They are highly susceptible to degradation by nucleases, have relatively low efficiency in targeted delivery to specific tissues, and the carriers used can provoke immunogenic responses [157]. Due to the presence of specific nucleases in serum, such as serum RNase A-type nuclease, the biological half-life in blood and cells differs significantly [158]. This problem can be overcome by chemical modifications of nucleotides, such as methylation or fluorination of the 2′ ribose [159].

To enhance delivery efficiency and protect miRNA from degradation, many specific carriers have been developed. Among them, lipid-based delivery systems are widely used [160]. However, their limited specificity makes targeted delivery to cardiac cells particularly challenging. Functionalization of liposomes has shown promising results. Attaching a specific peptide to their surface that binds to cardiomyocytes, particularly in areas of ischemia and remodeling, enabled targeted delivery of the miR-185-5p inhibitor. As a result, a significant improvement in EF and a reduction in fibrosis were achieved [159]. Another significant limitation is the low solubility and short half-life of liposomes. For this reason, the use of polymeric delivery systems such as polyethyleneimines (PEIs) has been explored. Due to their positive electrostatic charge, they strongly bind negatively charged miRNA molecules [161]. Due to their high cytotoxicity, clinical applications usually require combination with other polymers such as poly L-Lysine (PLL) or polyethylene glycol (PEG) [162,163]. Ease of aggregation and uncontrolled release of the transported substances are also problematic [161]. The use of extracellular vesicles (EVs) also offers hope for increased treatment efficacy. MiRNA-21 transported in this manner led to significant improvement in cardiac function in a preclinical MI animal model [164]. Nonetheless, the efficiency of miRNA loading into EVs is often inconsistent and low, and EV subpopulations exhibit distinct physicochemical and biological properties, which impacts the reproducibility of results and complicates dose control. Additionally, off-target uptake by phagocytic cells remains a significant problem [165]. The use of viral vectors, such as lentiviruses, adenoviruses, and adeno-associated viruses, is also popular. Unlike EVs, their significant advantage is high infection efficiency and greater specific tissue selectivity [166]. Injection of AAV9-anti-miR-199a reduced hypertrophy and increased ejection fraction in mice treated with isoproterenol [167]. Unfortunately, viral vectors exhibit relatively high immunogenicity, which may lead to damage to transfected cells and reduce the effectiveness of therapy due to binding to neutralizing antibodies [166]. In a phase 1/2 clinical trial in patients with HF, cases of AAV vector therapy failure due to the presence of the above-mentioned antibodies were observed [168].

Additionally, ultrasound-targeted microbubble cavitation (UTMC) is being explored. This technique involves the intravenous administration of lipid-protein vesicles filled with gas and a therapeutic and is followed by exposing the target tissue to ultrasound, which induces cavitation and cargo release. AntimiR-23a administered this way reduced cardiac hypertrophy in mice [169]. Local delivery of hydrogels containing polymer nanoparticles with miRNA has also been attempted. In a study of rats with myocardial infarction, administration of miR-199a-3p by this method resulted in an increase in ejection fraction and a reduction in scar size [170]. Unfortunately, the carriers themselves can also be toxic. Cationic liposomes, through electrostatic interactions with negatively charged membranes, destabilize them. Modifications that alter their surface charge, such as pegylation, reduce their cytotoxicity [171].

MiRNAs may be promising biomarkers for early disease detection. Currently, the main problem is the lack of standardization in analytical and preanalytical procedures. Measurements may not be reproducible due to low miRNA stability. Comparing studies becomes difficult due to the use of different protocols—miRNA levels are influenced by factors such as the type of anticoagulant, sample storage time, temperature, and the specific isolation method used [90].

### 5.2. Future Directions and Emerging Opportunities

Combining miRNA with other therapies can yield synergistic effects. Transduction of transplanted cardiac progenitor cells (CPCs) with miR-21, miR-24, and miR-221 after MI in mice increased their survival. Furthermore, improved left ventricular fractional shortening was observed compared to the group receiving unmodified CPCs. This effect is likely due to inhibition of Bim gene expression, a key activator of apoptosis.

The rapid development of artificial intelligence observed in recent years may contribute to significant progress in miRNA research. Neural networks can help predict miRNA-mRNA interactions, which contributes to improved sensitivity for noncanonical binding events [172]. A 2021 study using distinct machine learning approaches identified two circulating miRNAs that can aid in the diagnosis of pulmonary hypertension [173]. Thanks to increasingly advanced algorithms, a personalized multi-omics approach may become possible, where data based on miRNA levels, microbiome composition, and levels of specific metabolites will enable more precise diagnoses and prognoses [174].

Early attempts to detect miRNAs relied primarily on techniques such as PCR, which required the use of material obtained from multiple cells for a single measurement [175]. This made it difficult to determine cellular heterogeneity. The advent of single-cell miRNA sequencing (sc-miRNA-seq), which can provide information on miRNA populations in individual cells, was a breakthrough [176]. Importantly, integration with spatial transcriptomics allows not only the identification of biological processes in tissue but also the precise localization of these processes in specific tissue regions, enabling the creation of high-resolution maps of miRNA activity [177]. Currently, there is a lack of studies using AI and modern methods such as sc-miRNA-seq in pediatric cardiology. Further research is needed to implement these approaches in this field. Perhaps this approach will soon enable better assessment of cardiac tissue heterogeneity during development, mapping miRNA activity in regions affected by congenital defects, and better identifying potential biomarkers.

## 6. Conclusions

MiRNAs constitute a key regulatory link in heart development and proper function. They influence key cellular processes such as proliferation, differentiation, and apoptosis. A thorough understanding of the molecular mechanisms underlying these phenomena offers hope for the development of new diagnostic and therapeutic methods. In recent years, significant progress has been made in research on the use of miRNAs in the diagnosis and treatment of various conditions, such as congenital heart defects, arrhythmias, carditis, and heart failure in children. Unfortunately, research focused on their practical application in clinical medicine, including monitoring the course of cardiovascular disease, assessing therapeutic efficacy, and identifying new therapeutic targets, is still lacking.

## Figures and Tables

**Table 2:** Key microRNAs in congenital heart diseases. ASD—atrial septal defect, BAV—bicuspid aortic valve, HF—heart failure, HLHS—hypoplastic left heart syndrome, TAV—tricuspid aortic valve, TOF—tetralogy of Fallot, UAV—unicuspid aortic valve, RVOT—right ventricle outflow tract.

Disease	Key miRNAs	Expression Pattern	Clinical Significance and Function	References
HLHS	miR-150-5p	Downregulated in serum	Potential biomarker for HF, especially when combined with NT-proBNP	[7]
	miR-129-5p	Downregulated in serum	Potential biomarker for HF, likely associated with hypoxic response	[8]
TOF	miR-19b, miR-19c, miR-375, miR-22	Upregulated in maternal serum	Potential biomarker	[85]
	miR-421, miR-1201	Upregulated in RV tissue	Mediator of RV remodeling in TOF, miR-421, negatively correlated with SOX4	[98,99]
	miR625-5p, miR-183-5p	Upregulated in serum	Potential biomarker	[6]
	miR-181d-5p, miR-142-5p, miR-339-5p	Downregulated in serum	Potential biomarker	[6]
	miR-424, miR-222	Upregulated in RVOT tissue	Enhanced proliferation, additionally, miR-222 leads to inhibition of cardiomyocyte differentiation (murine models)	[100]
	miR-940	Downregulated in RVOT tissue	Enhanced proliferation and inhibition lead to reduced migration, acting through the inhibition of JARID2, a protein essential for outflow tract morphogenesis	[101]
	miR-1, miR-206	Downregulated in RVOT tissues	Imparing myocardial conduction—increased expression of Cx-43, disrupted expression of KCNJ2 and TNNI1	[6,102,103]
	miR-221-5p, miR-21-5p, miR-155-5p	Upregulated in serum	Potential biomarkers, mainly active in hypoxia-sensitive signaling pathways	[104]
	miR-21-5p, miR-144-3p	Downregulated in serum		
	miR-200a-3p, miR-10a-5p	Upregulated in amniotic fluid	Potential biomarkers, acting through inhibition of cardiomyocyte differentiation via suppression of the transcription factor GATA4, while miR-10a-5p additionally suppresses TBX5 and NKX2.5	[105]
VSD	miR-1-1	Downregulated in heart tissue	Inhibition of GJA1 and SOX9	[10]
	miR-181c	Upregulated in heart tissue	Downregulation of BMPR2	[10]
	hsa-let-7e-5p, hsa-miR-155-5p, hsa-miR-222-3p, hsa-miR-379-5p, hsa-miR- 409-3p, hsa-miR-433, hsa-miR-487b	Downregulated in serum	Impact on the expression of genes critical for cardiac development, including HAND1, NKX2-4, TBX1, MAP2K4, GATA4, NOTCH1 and ZFPM2	[111]
	hsa-miR-498	Upregulated in serum		
	miR-142-5p, miR-4666a-3p	Downregulated in maternal serum	Potential biomarkers	[86]
	miR-1275, miR-3664-3p	Upregulated in maternal serum		
	hsa-miR-146a-5p	Downregulated in maternal serum	Potential biomarker, acting through the suppression of NUMB expression, a negative regulator of the Notch pathway	[84]
ASD	miR-196a2	Collected from serum	SNP of this miRNA is associated with disease susceptibility	[112]
	miR-139-5p	Mutation identified by whole-genome sequencing	A mutation in the 3′UTR of the ACTC1 gene results in the de novo creation of a binding site for miR-139-5p and disease development	[113]
	hsa-let-7a, hsa-let-7b, hsa-miR-486	Upregulated in serum, hsa-let-7a also showed a positive correlation between levels in maternal and child serum	Potential biomarker in prenatal screening, miR-486 also exhibited significantly higher levels among patients with VSD and AVSD	[11]
	miR-29, miR-143/145	Upregulated in murine atrial septum tissue	Involvement in the regulation of multiple pathways essential for morphogenesis.	[114]
	miR-17-92, miR-106b-25, miR-503/424	Downregulated in murine atrial septum tissue		
BAV	miR-130a	Downregulated in serum	Potential biomarker, levels reduced in patients with BAV and dilated aorta, acting through modulation of the TGF-β pathway	[129]
	let-7e-5p, miR-196a-5p	Upregulated in aortic wall tissue	Changes in concentration relative to controls were particularly pronounced in groups with BAV and aortic dilation	[130]
	miR-17a-5p	Downregulated in aortic wall tissue		
	miR-21, miR-133a, miR-143, miR-145	Positive correlation between plasma and aortic tissue concentrations among BAV and UAV patients	Association between investigated miRNAs and aortopathies	[131]
	miR-424-3p, miR-3688-3p	Downregulated in aortic aneurysm tissue among patients with BAV compared to TAV	Modulation of the expression of genes such as Hippo, ErbB, and TGF-β involved in the development of the valvular apparatus	[132]
	miR-128-3p	Downregulated in the dilated segments of aortic tissue	Associated with inflammatory response pathways	[133]
	miR-150-5p, and miR-199b-5p	Upregulated in the dilated segments of aortic tissue	Related to eicosanoid synthesis and the regulation of VEGF signaling pathways	

## Data Availability

No new data were created or analyzed in this study. Data sharing does not apply to this article.

## Abbreviations

The following abbreviations are used in this manuscript:

ASD	atrial septal defect
Ago	Argonaute proteins
BAV	bicuspid aortic valve
BMP	bone morphogenic proteins
CPCs	cardiac progenitor cells
cTNT	cardiac Troponin T
CoA	coarctation of the aorta
CHD	congenital heart disease
Cx43	connexin 43
FGF	fibroblast growth factor
hESCs-CMs	human embryonic Stem Cells–derived Cardiomyocytes
HLHS	hypoplastic left heart syndrome
miRNA	microRNA
mRNA	messenger RNA
PDA	patent ductus arteriosus
PABPC	poly(A)-binding protein, cytoplasmic
PEG	polyethylene glycol
PEIs	polyethyleneimines
PLL	poly L-Lysine
Pol II	RNA polymerase II
RV	right ventricle
RVOT	right ventricular outflow tract
RISC	RNA-induced silencing complex
sc-miRNA-seq	single-cell microRNA sequencing
TDMD	target-directed microRNA degradation
TA	tricuspid atresia
TGA	transposition of the great arteries
TOF	tetralogy of fallot
TET2	Tet meyhylcytosine dioxygense 2
VSD	ventricular septal defect
